# High frequency Lunar Penetrating Radar quality control, editing and processing of Chang’E-4 lunar mission

**DOI:** 10.1038/s41597-024-02963-4

**Published:** 2024-01-24

**Authors:** G. Roncoroni, E. Forte, I. Santin, A. Černok, A. Rajšić, A. Frigeri, M. Pipan

**Affiliations:** 1 Department of Mathematics, Informatics and Geosciences, University of Trieste, Trieste, Italy; 2 Department of Earth, Atmospheric and Planetary Sciences, Purdue University, West Lafayette, Indiana USA; 3 Istituto di Astrofisica e Planetologia Spaziali (IAPS), Istituto Nazionale di Astrofisica (INAF), Rome, Italy

**Keywords:** Planetary science, Databases

## Abstract

Chinese lunar landing mission Chang’E-4 reached the far side of the Moon in January 2019 and has been providing unprecedented Lunar Penetrating Radar data able to explore the lunar subsurface down to more than 40 m (with its more resolutive high frequency band). Data are periodically released to the scientific community in raw PDS4 format. Here we provide different versions of the radar dataset after editing (i.e. pre-processing), partial, and full processing in order to provide a complete ready-to-use dataset to end-users (data collected since 4^th^ January 2019 until 27^th^ March 2023) which can be directly exploited for analysis, interpretation, inversion, as well as integration with imagery or other information. In particular, we implemented an efficient and objective way to remove duplicated traces representing more than 90% of original data, as well as a processing flow able to retain all the original data information, while avoiding redundancies. The provided datasets can be implemented with future data releases and straightforwardly exploited for any future analysis.

## Background & Summary

The Chinese lunar landing mission Chang’E-4 (CE-4) landed on January 3^rd^, 2019, in the ancient Van Kármán crater (diameter D = 185 km; 177.5991°E, 45.4446 °S), located on the far side of the Moon^1^. CE-4 mission follows the previous CE-3 mission, which aimed at the exploration of the lunar subsurface structures through a Lunar Penetrating Radar (LPR) while analysing the mineralogical composition by collecting *in situ* reflectance spectra and taking panoramic photographs. To achieve this, the Yutu-2 rover^2^, of the CE-4 mission, is equipped with a dual frequency LPR, with central frequencies equal to 60 and 500 MHz (respectively CH-1 and CH-2), among several other sensors. LPR instruments is almost identical to Ground Penetrating Radar (GPR) devices commonly adopted as a near surface high-resolution geophysical tool on the Earth’s surface. The low-frequency data of the LPR system are affected by interference phenomena first described for the CE-3 mission^3^ and then reported also for that of the CE-4^4^. Our work focuses on the high-frequency LPR dataset because of its high quality and potential information content emerged from several preliminary analyses e.g.^2,5^.

The fundamental goal of the LPR survey is to investigate the lunar subsurface along the rover’s path down to several tens or even hundreds of meters^6–8^, with horizontal and vertical spatial resolutions of ~0.1 meter. Since landing, the rover has been moving along an irregular path, segmented into sectors separated by many stops and turnaround points. The initial studies focused on the first hundreds of meters of the path by applying different analysis, processing, and inversion algorithms^8,9^, with the main aim of improving data interpretation^6–10^. LPR data are publicly released through moon.bao.ac.cn/ website with a delay to the acquisition, due to an embargo period. Up to now (July 2023) a total of 634,419 A-scans (i.e. traces) for a total length of the path equal to ~1440 m, within the SOL range (i.e. each single data file identification number) between 01 (4^th^ January 2019) and 286 (27^th^ March 2023) for a total of 160 SOL files, have been released.

**Fig. 1:**
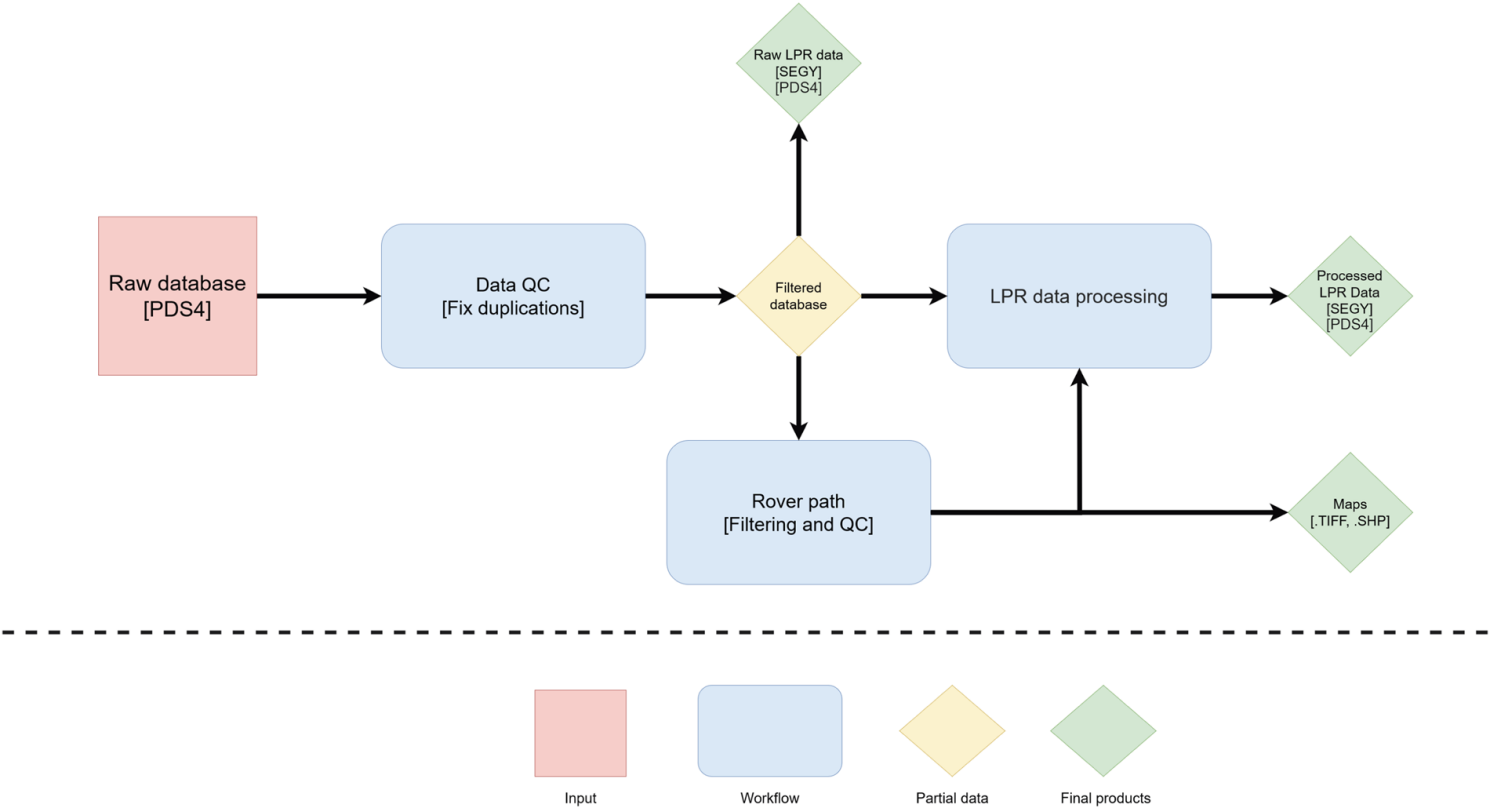
PDS4 database (input), in red; workflow of the methodology, in blue; available dataset from the current work (outputs), in green. QC refers to the quality control steps applied to the data.

Our methodology aims at providing a standard workflow to get PDS4 and SEG-Y (IBM float 4 bytes) edited and processed LPR data ready for the interpretation process, starting from the original dataset release in separated PDS4 format files (Fig. 1). While PDS4 is a format used primarily by NASA to store and distribute solar, lunar and planetary imagery data (https://pds.nasa.gov/datastandards/documents/), SEG-Y is the standard format developed by the Society of Exploration Geophysicists (SEG) to store reflection seismic geophysical data^11^ (http://seg.org/Publications/SEG-Technical-Standards). Raw database files contain both spatial information (e.g. rover path coordinates) and the raw LPR data, which need to be filtered and analysed for quality assessment and usage. Before applying any processing algorithm, it is essential to edit the data. In fact, the rover collects LPR A-scans continuously, even when it stops to acquire other data. All such repeated redundant scans must be properly recognized and removed during a dedicated pre-processing (i.e. editing) phase.

The LPR dataset is then processed considering a basic and standard GPR processing flow integrated by surface information about the rover path, which was essential to perform topographic correction and identify artifacts on the LPR data. Furthermore, the rover path is not straight and it often has an almost duplicated route going back and forth along two similar trajectories. This reflects on the data with a peculiar symmetrical behavior with respect to the turnaround points. Identifying these artifacts is fundamental to avoid misinterpretation of the data^12^.

A standard workflow from data download to quality control and data processing is an essential tool in order to make data analysis and interpretation reliable and objective. The ultimate goal is to improve the interpretation and comprehension of lunar subsurface structures, which in some cases were not previously imaged and properly considered. Signal data processing is in fact essential for all the GPR datasets to make possible a correct subsurface imaging and to estimate the electromagnetic physical parameters. In this light, the edited and ready-to-analyse datasets (Fig. 1) provided together with this article can represent a crucial starting point for future studies based on CE-4 data, but also on other integrated lunar analyses. All the data will be possibly integrated when further releases will be made available by the National Astronomical Observatory of the Chinese Academy of Sciences.

## Methods

### LPR data pre-processing and editing

Prior to the processing flow, that is usually performed on any GPR data acquired on the Earth, we considered the peculiar issues related to duplicated traces and data file stitching^13^. Considering the acquisition set up, the rover makes several stops to acquire other measurements during its movement, e.g. panoramic cam or visible near infrared spectroscopy, during which it does not interrupt the acquisition of LPR data. As a consequence, most of the raw data are redundant and must be removed. Since the accuracy of recorded coordinates is not high enough (see section Rover Coordinates), we have designed an algorithm capable of automatically recognizing and removing duplicated scans, minimizing the subjectivity of the procedure, saving time, and avoiding possible residual duplications (the complete code is freely available at https://github.com/Giacomo-Roncoroni/LPR_CE4/).

**Fig. 2:**
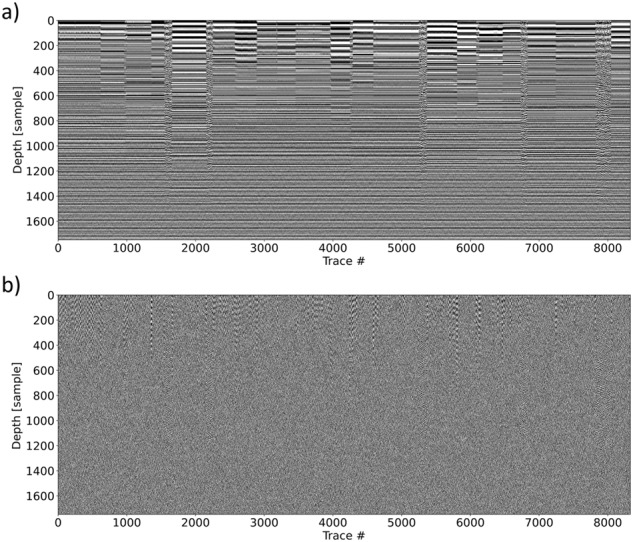
SOL2 after de-wow (**a**) and Sobel filter (**b**).
- Apply a Sobel filter in x direction with a kernel size of 5, in order to suppress signals from the zones where the rover was not moving (Fig. 2b).
- Apply a median filter to reduce spikes and random noise in the dataset.
- Compute trace energy, i.e., sum the absolute values of the filtered traces along the time axis.
- Select just the energy values, within a set window (16 traces), that are over a threshold value (25000), kept constant for the whole dataset. These values were selected by a grid search on the parameters on the data; slightly different threshold values indeed produce almost identical outputs.

The proposed workflow summarized and described here is therefore implemented to reduce unwanted features, while retaining just the actual information contained into the dataset:

- Open separated PDS4 files selecting all LPR data and coordinates.
- Apply a de-wow, i.e., removing the first 300 samples of the dataset, due to the presence of clipped amplitudes (Fig. 2a).

**Fig. 3:**
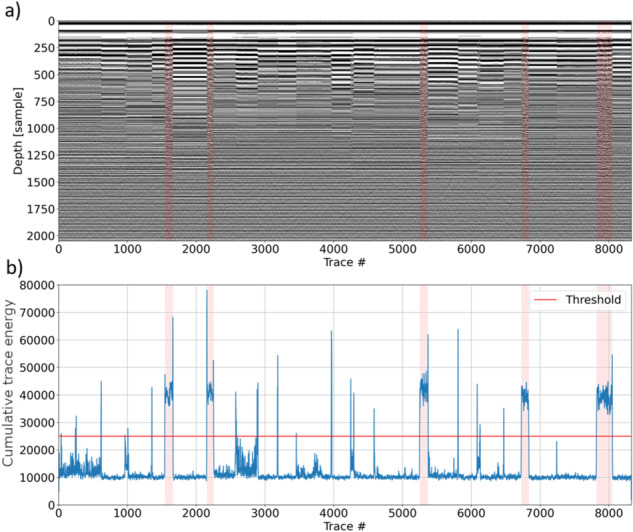
Portion of raw data (**a**) and a corresponding energy plot (blue curve), set threshold (red line), (**b**). Selected scans related to the moving rover are marked in light red.

The whole procedure was first applied to identify the proper cutting of the limits/intervals, then we reapplied the workflow considering these intervals on the unprocessed data in order to not lose of information due to the Sobel filter (Fig. 3). Furthermore, the same windows and thresholds set above were applied also to data collected during the movement of the rover. The same procedure was applied to the entire dataset.

**Fig. 4:**
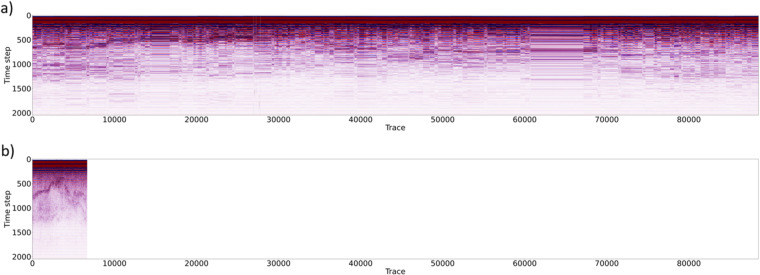
Last 15% of the original dataset before (**a**) and after (**b**) redundant A-scans removal. Some subsurface reflectors are apparent in (**b**) while they are not recognizable in (**a**).

As mentioned above, data acquired in different lunar days are stored separately in different files (SOL) and need to be merged to get a manageable full dataset. From 634,419 A-scans stored in the original SOL files, after the duplication removal we obtained a 40,022 A-scans long LPR B-scan (i.e. profile). Figure 4 shows the reduction of redundant information on a portion of the dataset (from a to b) and the unprocessed data obtained after the duplicated scans removal (b). From Fig. 4b we can see the high quality of the data on which some subsurface structures are already apparent.

### Rover coordinates

**Fig. 5:**
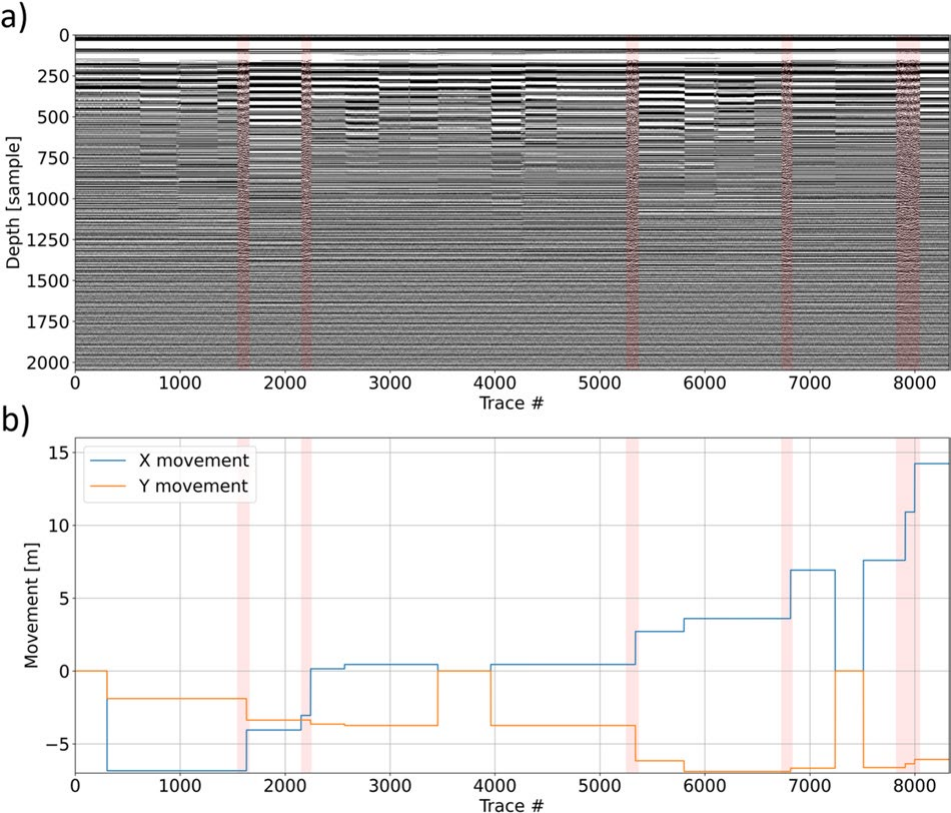
LPR data (**a**) and the corresponding stored relative movement (extracted from the stored PDS4 coordinates) of the rover (**b**). Identified areas of actual movement (as in Fig. 3) are marked in light red.

LPR data stored are not the only data affected by data redundancy and the other issues highlighted above: also acquisition coordinates are affected in the same way. If the stored coordinates were accurate enough, they could be used to select and retain only the scans associated to the actual rover movement, but this is not the case (Fig. 5). In fact, there are zones in which the coordinates change implying a rover movement which, however, did not take place considering that the A-scans recorded at these locations are indeed identical (e.g. near traces 300 or 7500).

**Fig. 6:**
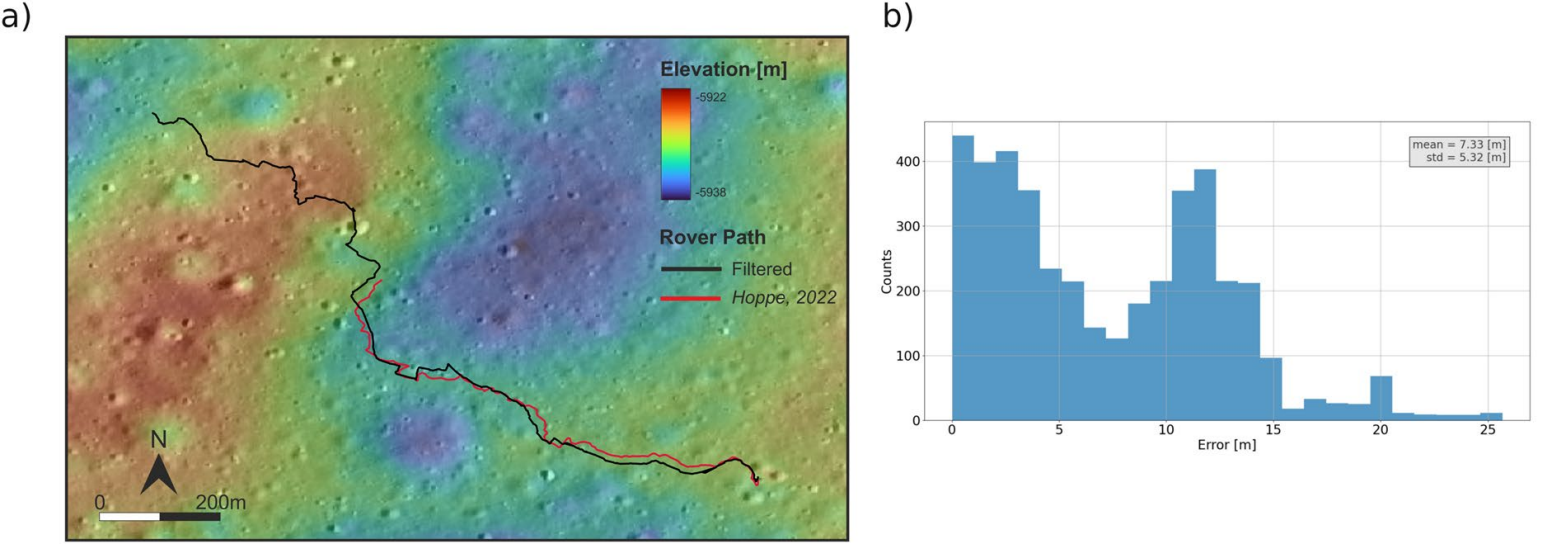
Rover path extracted following our procedure (in black) and the one derived in Hoppe, 2022, (**a**) and histogram of the differences between the two estimated paths, (**b**).

Figure 6 compares the rover path extracted from the data filtered as described above and the one provided by Hoppe, 2022 (available at http://lroc.sese.asu.edu/posts/1248). It can be seen that the main mismatch occurs at the beginning and at the end (for the red line) of the rover path. The mean error is equal to 7.33 m with a standard deviation equal to 5.32 m.

Knowing the rover path details is essential to better discriminate evidence of actual subsurface structures on the data, avoiding interpreting signals which are just due to rover turnarounds and repeated paths. Information about data positioning allowed us to georeference the LPR data in the absolute location.

As the rover movements information in original PDS4 files are provided in meters, we decided to reproject the rover path on a metric reference system with an origin that corresponds to the CE-4 landing site, always assuming a constant A-scan distance for each selected portion of actual movement. As a result, we reprojected to our custom reference system also the orthophoto and the Digital Elevation Model (available at https://quickmap.lroc.asu.edu/). This latter step is mandatory for data integration and correlation of features evidenced on different datasets and imagery.

### LPR data processing

**Fig. 7:**
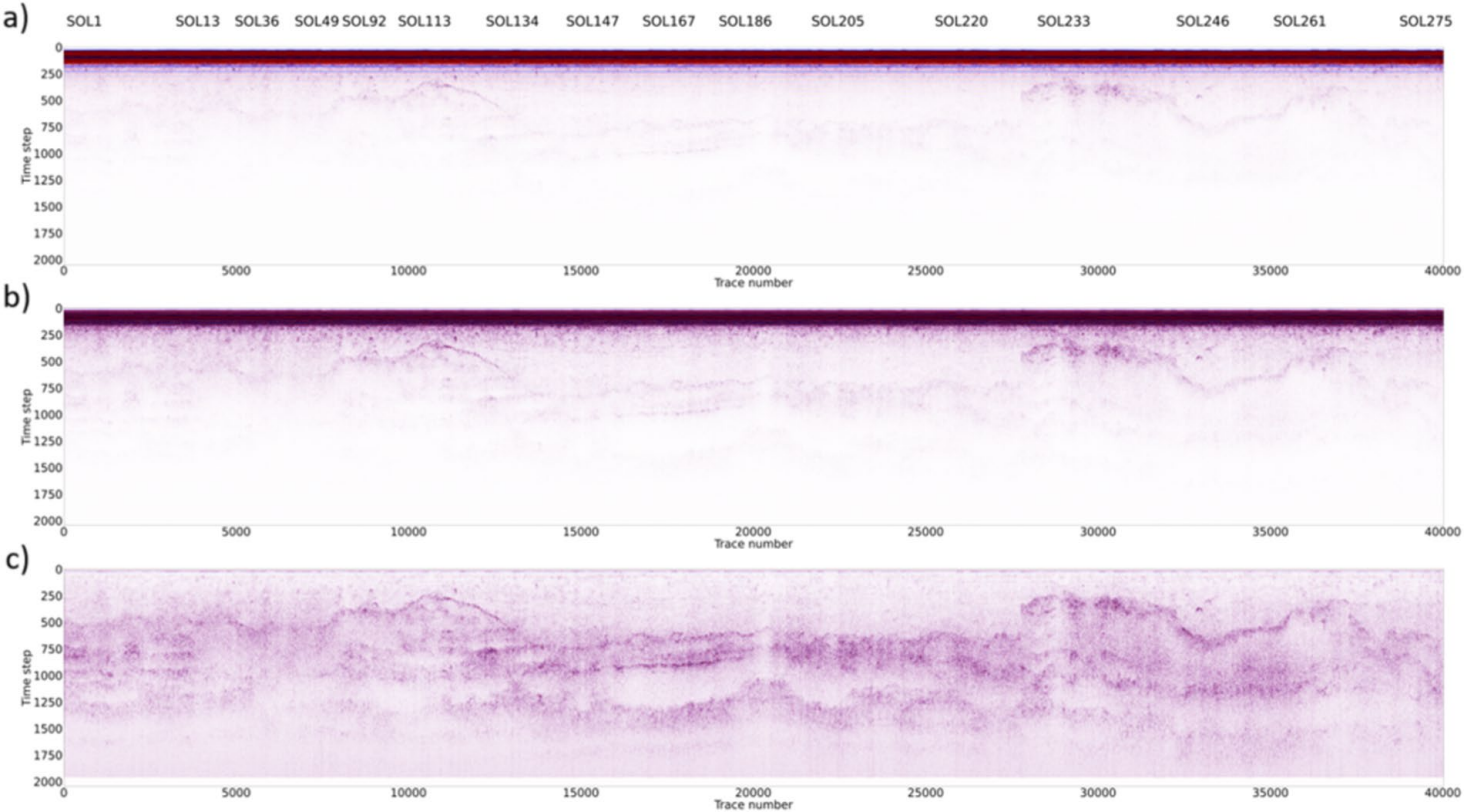
Effects of the processing workflow applied on the whole dataset: raw data (**a**); data after the zero-time correction and bandpass filtering (**b**); data after horizontal high pass and gain in addition to the previous steps (**c**).

Once the entire CE-4 dataset was edited (Fig. 7a), we applied a very conservative processing workflow to preserve the information related to amplitude and spectral content, both crucial for further data interpretation and analysis^14^.

The proposed processing workflow is performed in python and a step-by-step description and parametrization can be found at https://github.com/Giacomo-Roncoroni/LPR_CE4/tree/main/01_LPR_processing.

**Fig. 8:**
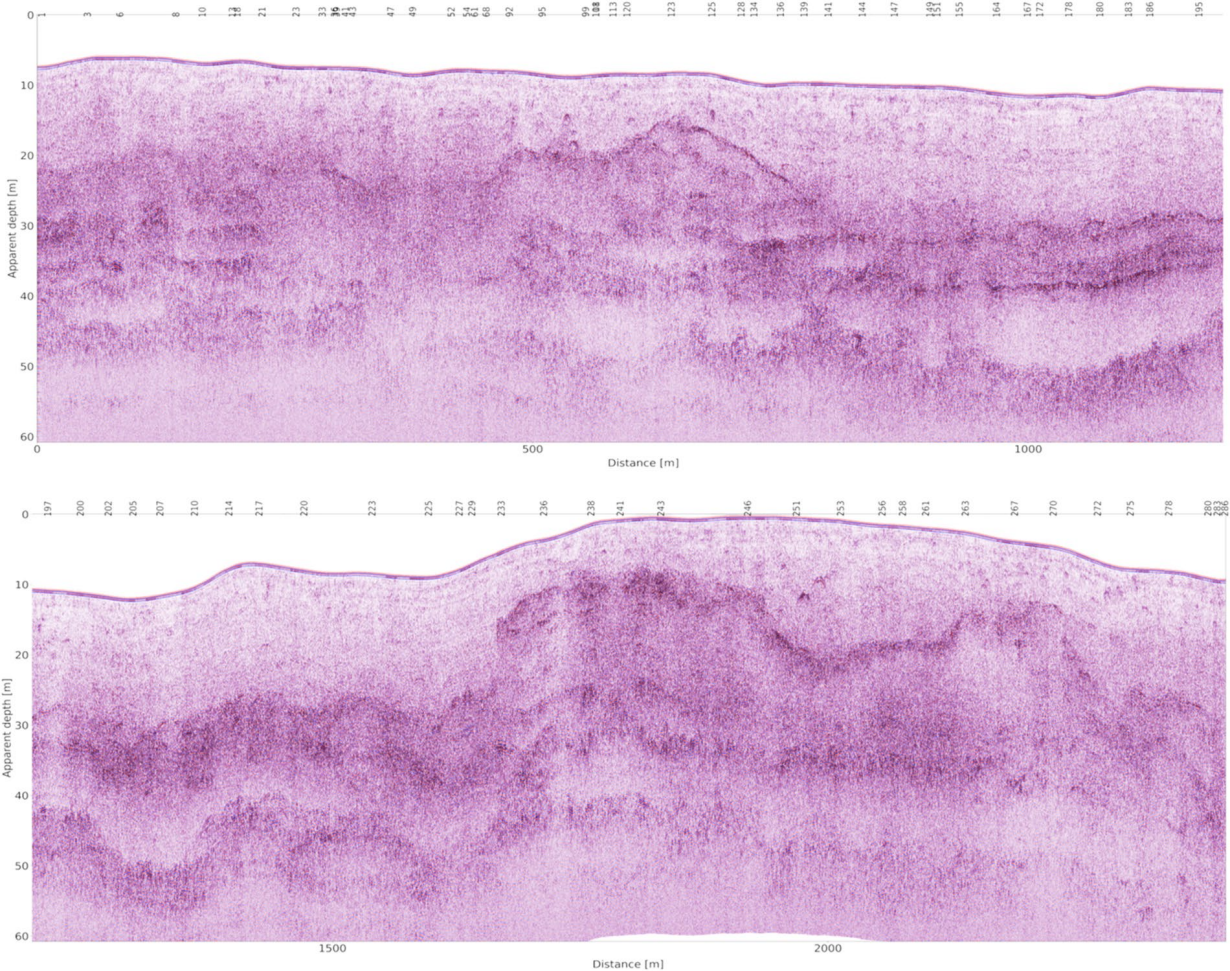
Final processed CE-4 LPR dataset (split in two separate sections just for a better visualization) obtained after the application of the complete processing flow described in the text.

The proposed workflow^14^ is based on five main steps:

- Bandpass filter
- Zero-time correction (drift removal), Fig. 7b
- Horizontal High pass filter (background removal)
- Exponential amplitude compensation (gain), Fig. 7c
- Static (topographic) correction and depth conversion, Fig. 8

We applied a time to depth conversion with a constant velocity equal to 0.16 [m/ns], which is in good agreement with the most recent estimates^15,16^. Other different non-constant estimates are available in literature, but their reliability and accuracy are still debated. The final processed data including static correction, i.e. correction of the position in the z direction applied, is shown in Fig. 8. Elevation data were derived by the Digital Elevation Model available on https://quickmap.lroc.asu.edu/ considering A-scans with constant 0.036 m trace spacing^17^. The zero reference (datum) represents the maximum elevation of the rover along the considered path.

## Data Records

The raw dataset is available in PDS4 format in Figshare^18^ and in SEG-Y format in Figshare^19^,maps and rover path are available in Figshare^20^.

As shown in Fig. 1, the data resulting from our workflow are both edited and processed CE-4 LPR data, as well as their filtered spatial coordinates.

LPR data are stored as standard PDS4 and SEG-Y formats files with spatial information already saved in the file headers XPOSITION, YPOSITION and at bytes 73 and 77, respectively.

Specifically, the released PDS data are split in two files with extensions: 0.2BL for the text headers and 0.2B for the binary data files. The three datasets are:

- CE4_RAW_LPR_CH2_20190104_20230327_0000 → the edited data version, just filtered from the redundant repeated data.
- CE4_PROCESSED_LPR_CH2_20190104_20230327_0001 → the processed data version without the static (topographic) correction.
- CE4_PROCESSED_STATIC_LPR_CH2_20190104_20230327_0002 → the processed depth converted data version with static correction applied.

The three SEG-Y datasets are:

- 00_moon_final_raw → the edited data version, just filtered from the redundant repeated data.
- 01_moon_final_proc → the processed data version without the static (topographic) correction.
- 02_moon_final_proc_static → the processed depth converted data version with static correction applied.

Coordinates files are store as text files (.txt) and are relative to the landing site (having 0, 0 coordinated): positive y are northwards and positive x are eastwards.

We also include the orthophoto and the DEM also centered in [0, 0] at the landing site.

## Technical Validation

**Fig. 9:**
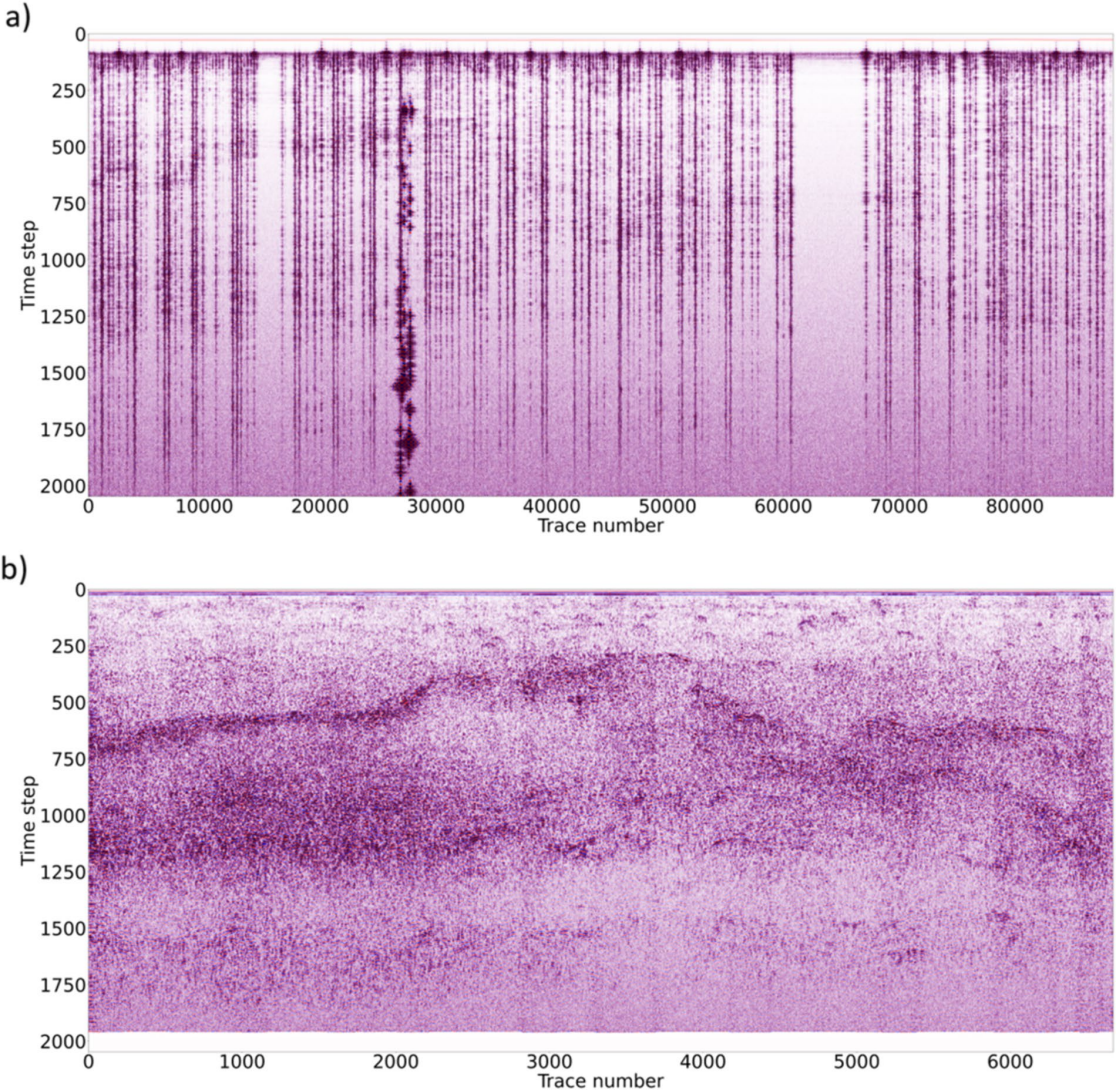
Processing workflow applied to the complete original (redundant) dataset (**a**) and to the edited dataset after removal of all redundant records (**b**).

In order to prove the validity of the methodology, we applied the previously described processing methodology both on original and filtered data (Fig. 9). As we can see in Fig. 9a, it is impossible to interpret the original dataset due to data redundancy, while Fig. 9b shows filtered data in which several reflectors can be easily identified.

**Fig. 10:**
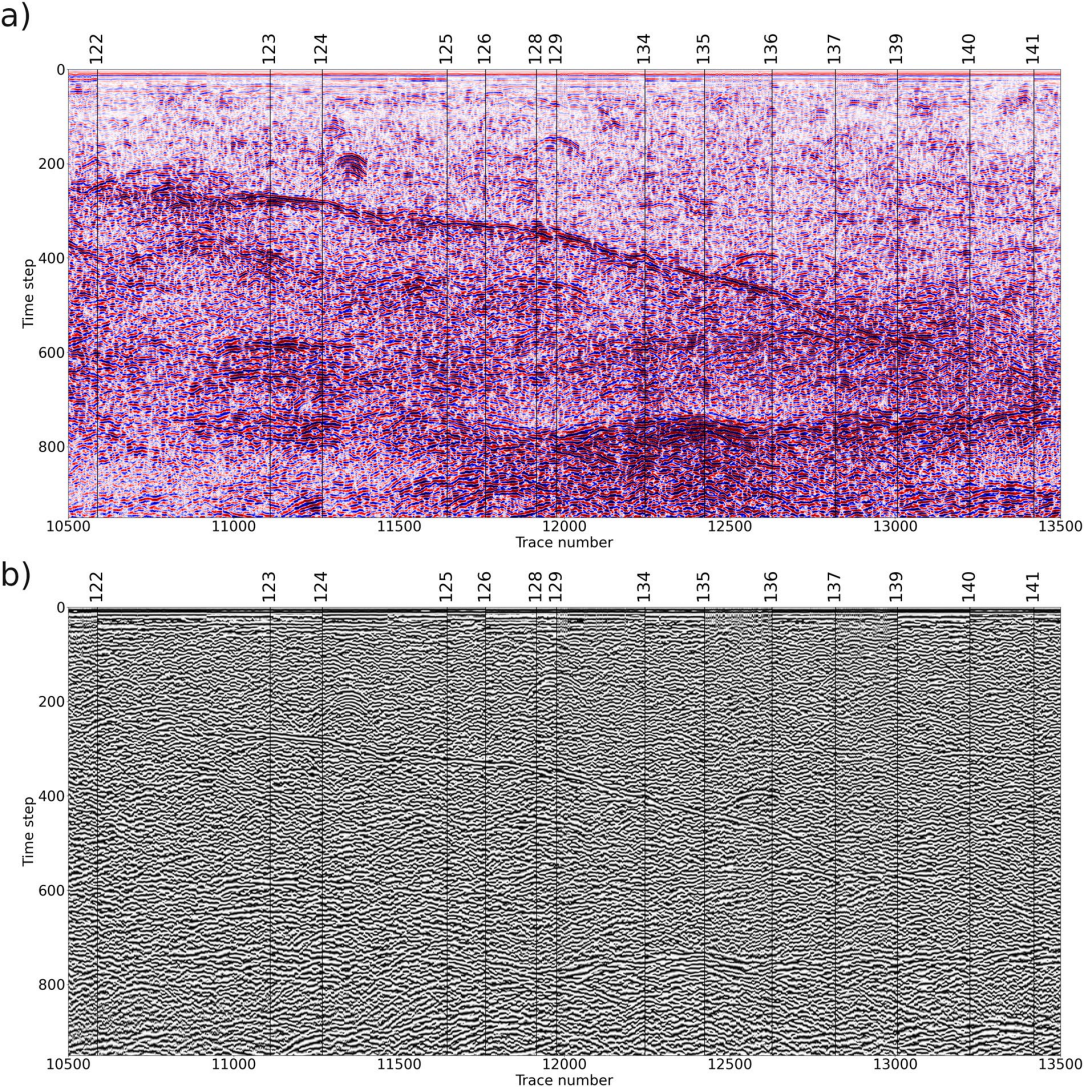
Test on lateral continuity on an exemplary portion of the edited and processed dataset with superimposed the SOL numbers as for the original released data. (**a**) signal amplitude data; (**b**) cosine of instantaneous phase data.

Moreover, in order to check the performances of the methodology, in Fig. 10 we highlight that the lateral continuity of the tilted reflector is guaranteed after the application of our methodology, even at the connection between originally separated data files, marked with vertical black lines in Fig. 10. In particular, we checked the signal phase behavior by calculating the cosine of the instantaneous phase (see e.g.^21^) which already demonstrated its capability to highlight signal phase discontinuities, even when GPR signals are characterized by similar amplitudes^22^.

SEG-Y data provided in this paper are tested to properly work on different commercial and open-source programs namely: ProMAX (Halliburton), Petrel 17 (Schlumberger), Seisee 2.22 (Dalmorneftegeofizika Geophysical Company), Prism 2.70.04 (Radar Systems), Reflex 9.5.7 (Sandmaier). PDS4 data have been properly opened using PDS4_tools (see https://github.com/Giacomo-Roncoroni/LPR_CE4/tree/main/03_create_pds4).

## Usage Notes

A summary of the main data parameters is presented in Table 1. This data descriptor was peer reviewed in 2023 based on the data available on the platform at the time.

**Table 1:** Summary of the main data parameters.

Variable	Value	Units
Number of traces (A-scans) retained after the redundant traces removal	40022	[adimensional]
Number of time samples per trace	2048	[adimensional]
Nominal signal central frequency	500	[MHz]
Time sampling interval	0.3125	[ns]
Trace distance	0.036	[m]
EM velocity used for static correction and depth conversion	0.16	[m/ns]

## Data Availability

The codes for the described algorithm will be available in Figshare^23^ and at https://github.com/Giacomo-Roncoroni/LPR_CE4.
